# Developing and Implementing a Colorectal Cancer Screening Program in Uganda: Stakeholder Perceived Barriers and Opportunities

**DOI:** 10.1002/cam4.70662

**Published:** 2025-03-13

**Authors:** Nicholas Matovu, Helen G. Coleman, Gerald Mutungi, Michael Donnelly, Lynne Lohfeld, Brian T. Johnston, Maurice B. Loughrey, Noleb M. Mugisha, Charlene M. McShane

**Affiliations:** ^1^ Centre for Public Health Queen's University Belfast Belfast UK; ^2^ Patrick G Johnston Centre for Cancer Research Queen's University Belfast Belfast UK; ^3^ Department of Non‐Communicable Diseases Ministry of Health Kampala Uganda; ^4^ Deartment of Gastroenterology Belfast Health and Social Care Trust Belfast UK; ^5^ Department of Cellular Pathology Belfast Health and Social Care Trust Belfast UK; ^6^ Uganda Cancer Institute Kampala Uganda

**Keywords:** colorectal cancer, global health, implementation, screening, stakeholder perspectives, Uganda

## Abstract

**Introduction:**

Colorectal cancer (CRC) incidence is increasing in Uganda. Despite this, and the disproportionately high burden of early onset and late‐stage CRC cases, no CRC screening program exists in Uganda. To guide and inform future CRC prevention efforts, interviews with key stakeholders were undertaken to better understand the perceived barriers and opportunities relevant to the development and implementation of a CRC screening program in Uganda.

**Methods:**

Semi‐structured key informant interviews were conducted with key stakeholders in cancer prevention, screening and policy/programming (*n* = 11, 6 medically qualified and 5 non‐medical), who were recruited across Uganda using maximum variation sampling between March and April 2022. Interviews were audio recorded, transcribed, coded and later analysed using a deductive thematic analysis approach guided by the social ecological model.

**Results:**

Major barriers included lack of government priority for CRC prevention programs, lack of resources/funding for CRC screening (policy level), inadequate screening facilities and equipment, limited training/knowledge of CRC and capacity of the health workforce (health system level), challenges in the delivery of CRC awareness messages (community level), emotions associated with CRC screening and poor awareness of CRC and its symptoms (individual level). Major opportunities included the existence of a draft national cancer control plan (policy level), existence of less costly CRC screening alternatives, less costly primary prevention measures (health system level), existence of community leadership and structures (community level), likely acceptability of the faecal occult blood test and peer support (individual level).

**Conclusion:**

There are substantive barriers to CRC screening program development and implementation in Uganda. However, there are signs, like the development of a cancer control plan, that suggest a shift towards strategic planning and allocation of resources at a population level for addressing the issues of cancer prevention and care, including CRC. In the meantime, efforts should prioritise primary prevention interventions such as mass education to promote CRC awareness.

## Graphical abstract

Qualitative semi‐structured interviews were held with stakeholders in Uganda with responsibilities for delivering cancer prevention, screening and policy/programming to elicit their views and improve understanding regarding barriers to, and opportunities for, introducing a colorectal cancer screening program. Results indicated significant barriers across policy, health system, community and individual levels whilst noting opportunities to overcome these challenges, such as the drafting of the first national cancer control plan. Key priorities include colorectal cancer primary prevention (e.g., mass awareness raising) and using implementation science to embed evidence‐informed colorectal cancer screening in Uganda.

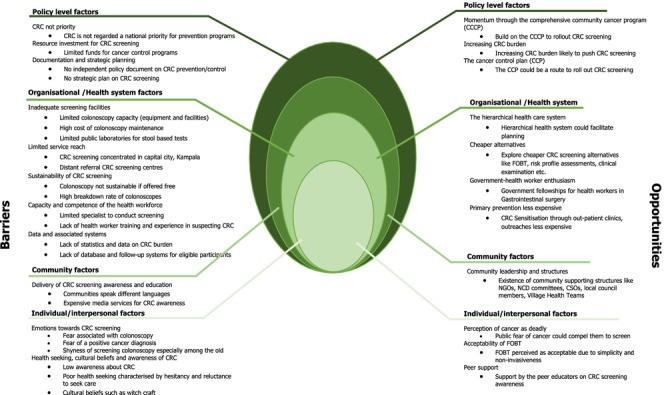

## 1 Introduction

While colorectal cancer (CRC) incidence rates are lower in low‐ and middle‐income countries (LMICs), mortality is considerably higher when compared to high‐income countries (HICs) [1]. Within Africa, CRC incidence is increasing [2], with a relatively high proportion of early onset [3] and late‐stage CRC being diagnosed [4] compared to countries in the Western world. Survival rates on the continent remain poor, with Uganda registering the lowest 5‐year relative survival at 5.6% (for all stages combined) in a recent study among 11 Sub‐Saharan African countries [4]. These figures are in stark contrast to 5‐year CRC relative survival rates from several high‐income countries (i.e., Australia, UK, Canada, Denmark, Ireland, New Zealand and Norway) that ranged between 59% and 71% [5] for all CRC stages. This could be partly attributed to the existence of population‐based CRC screening programs in these countries. Screening for CRC offers the opportunity both for early detection of cancer and for the removal of pre‐cancerous polyps, preventing their progression to CRC. The success of this approach is demonstrated by the fact that European countries with the longest standing CRC screening programs have the largest decreases in CRC incidence and mortality, according to a 2021 population‐based study [6].

Despite the proven benefits of CRC screening, interventions to screen for CRC have not been commonly implemented in LMICs, particularly in Africa [7], with only Morocco having piloted a demonstration project in 2018 [8]. Screening programs are complex and labour‐ and resource‐intensive, requiring ongoing monitoring, evaluation and treatment for those identified as having the disease. Therefore, many LMICs are likely to face challenges in implementing and sustaining such programs [7]. However, as part of the initial steps of introducing any screening program, the World Health Organisation (WHO) emphasises the need for mapping and engaging appropriate stakeholders, as well as conducting a situation analysis to ensure that the introduction of a screening program is the right course of action compared to other prevention strategies such as early diagnosis or awareness creation [9].

According to the 2022 Globocan data, the age‐standardised CRC incidence and mortality rates in Uganda were estimated at 7.3 and 5.5 cases per 100,000, respectively [1]. Analysis of CRC trends based on data from the Kampala cancer registry between 1991 and 2015 revealed that the incidence of CRC increased by 1.7%–2.2% annually [10]. A global burden of disease study conducted in 2022 showed that the CRC mortality rate in Uganda has increased by 10% from 2010 to 2019, which ranks Uganda among the top 13 countries (out of 54) in Africa with the highest increase in CRC age‐standardised mortality rate within that period [2].

Considering the relatively lower incidence rate of CRC in Uganda compared to other regions, establishing a population‐based screening program according to the Wilson and Jungner criteria [11] may not currently be justified. However, the number of CRC cases in Uganda is projected to increase by approximately 140% by 2045 [12]. Prevention and early detection will be crucial in mitigating the impact of the CRC burden and severity on the healthcare system.

Cancer prevention and control in Uganda, including for CRC, has been mainly driven by the Comprehensive Community Cancer Program at the Uganda Cancer Institute (UCI), which is the national institution responsible for cancer control in Uganda and a designated centre of excellence for oncology in East Africa. Through this program, there has been implementation of cancer prevention activities, including screening, at the UCI and through several outreaches; however, most of these activities have historically focussed on cervical and breast cancer. Relatively little attention has been given to less common but increasing cancers such as CRC. To lay the foundation for CRC prevention and control in Uganda, this study aimed to explore perceptions of various cancer prevention or screening experts on barriers and opportunities related to developing and implementing a CRC screening program in Uganda.

## 2 Methods

### 2.1 Study Design, Setting and Population

This was a qualitative descriptive study conducted among key institutional stakeholders involved in cancer prevention, control and screening in Uganda. This included representatives from the Ministry of Health, regional and district hospitals, primary health care centres, screening and endoscopy centres or units (both public and private), civil society bodies and philanthropic organisations engaged in cancer prevention or screening, and academic institutions across all regions of Uganda. As such stakeholders have a key role in policy, programming and screening of cancers, they have extensive knowledge and experience (drawn from other cancer screening initiatives) regarding probable barriers and opportunities for developing and implementing a future CRC screening program.

### 2.2 Eligibility

The inclusion criteria included individuals aged 18 years or older, having significant experience in designing, implementing, conducting, monitoring and/or evaluating cancer prevention, control or screening programs at the community, health facility and/or policy level in Uganda; the ability to speak English or the local language ‘Luganda’ fluently; the ability to provide written informed consent and being able to take part in a one‐time individual semi‐structured interview. Participants not fulfilling the aforementioned criteria were excluded.

### 2.3 Participant Recruitment and Sampling

A comprehensive scanning exercise that comprised searching available electronic and written sources such as websites, reports, blogs and newspapers for potential key informants, in addition to the identification of potential individuals (based on prior knowledge) was carried out by the first author (NM) with the help of two researchers (GM, NMM). Maximum variation sampling was undertaken to purposively select a range of key informants in the areas of cancer control, prevention and screening working at different levels of specialty and operation across Uganda (Table 1). Maximum variation sampling was chosen to ensure a multidisciplinary representation of cancer prevention, control and screening experts to capture diverse perspectives within this study. This enabled us to ascertain how different stakeholder perspectives on barriers or opportunities for developing and implementing a future screening program varied across different stakeholder specialisations and levels of operation. A stakeholder analysis, which is a process of identifying, assessing and prioritising stakeholders with a vested interest in a project, was conducted by NM, CMcS and HGC. Here, all probable stakeholders were listed and ranked for suitability of recruitment based on their presumed interest, influence and impact on the study. Where a nominated key informant/stakeholder chose not to participate, they were asked to nominate a suitably experienced colleague within the same institution/speciality.

**TABLE 1:** Selection of key stakeholders by field of specialisation and level of operation in Uganda.

Broad field	Speciality	Number and level of operation of key informants recruited
Designing, monitoring or evaluating cancer control policies or programs	Cancer program and policy specialists (non‐medical)	Total = 3 (Civil society organisation = 1) (Philanthropic organisation = 2)
Implementing cancer control programs	Cancer prevention/early detection specialists (non‐medical)	Total = 2 (National = 1) (District = 1)
Implementing cancer screening	CRC screening specialists (medical)	Total = 6 *Specialisations*: Gastroenterologists = 1 Oncologists = 1 Gastrointestinal surgeons = 2 Gastrointestinal pathologists = 1 Senior medical officers = 1

### 2.4 Theoretical Perspective

This study was guided by the theoretical perspective of the social ecological model (SEM) [13] which posits that various factors exert influence on individual behaviour and health outcomes by interacting at multiple levels, including individual, interpersonal, organisational, community and policy. With respect to this research project, different barriers or opportunities may be observed during program planning and implementation at several levels of the SEM. This model was chosen because it helps to understand how broader social and environmental factors interact with individual choices, guiding more effective interventions and policies that influence multiple levels simultaneously. The SEM has been widely applied in similar research [14] and service projects [15] to explore multi‐level factors and their influence regarding CRC screening program implementation and design.

### 2.5 Data Collection

A rapid literature review of studies on the barriers and opportunities for implementing a CRC screening program was conducted to inform the development of a bespoke semi‐structured interview guide tailored to the Ugandan context (see Appendix S1) [16, 17, 18, 19, 20, 21]. Potential participants were contacted by a member of the research team by email or telephone to introduce them to the study. Individuals expressing interest received the study information sheet and consent form by email. They were asked to return the signed consent form if they agreed to participate and identify a suitable time for the interview and the preferred interview mode (face‐to‐face at their workplace or virtually by telephone or video call). In total, 21 stakeholders were contacted for participation in the study, 11 of whom were interviewed (10 declined to participate mainly citing competing priorities such as clinical duties, see detailed demographics of non‐participants in Appendix S1). Before each interview, verbal consent was sought from the participants to record the interview. Interviews were conducted by the same researcher (NM) between March and April 2022 and lasted approximately 20–50 min (average 35 min). Handwritten field notes were also taken. All recorded interviews were in English and later transcribed verbatim after removing all identifying information by the interviewer to create Microsoft Word transcripts.

### 2.6 Data Analysis

An iterative approach to data collection and analysis was used whereby the interviewer (NM) in consultation with other team members (CMcS and HGC) went back and forth in cycles of data collection and analysis. This helped ensure that thematic data saturation had been achieved after the 11th interview. Thematic saturation is a point at which no new insights are obtained from additional data collection/analysis. Thematic saturation was opted for because the study sought to capture the full complexity of participant perspectives across all SEM‐informed themes. Evidence of this was that all the SEM‐informed themes were adequately represented in the dataset that spanned the specialties of the interviewed stakeholders [22].

Data were managed by creating tables in Microsoft Excel. Analysis was via the thematic analysis methodology as outlined by Braun and Clarke [23], where common patterns of meaning or themes identified in the data were systematically organised. Two researchers (NM, CMcS) completed the initial steps of analysis by independently familiarising themselves with the data through reading each transcript in‐depth multiple times and making notes on key points before identifying and labelling relevant passages with codes. An initial set of agreed‐upon codes was produced and recorded along with a brief definition in a codebook that was updated as needed and applied to each transcript. The analysts met face‐to‐face weekly during this process to compare codes and discuss any new codes identified. Through discussion, any identified discrepancies in codes were rectified by reaching a consensus and involving an experienced qualitative researcher (LL). The overall approach to the thematic analysis was deductive, where codes were grouped according to the levels of the SEM. Subthemes were then constructed within each main theme category.

To ensure rigour, relevant steps to meet the criteria proposed by Lincoln and Guba [24] were employed. This included carefully following the study protocol, working with an experienced qualitative researcher (LL), triangulation (multiple researchers with distinct disciplines or fields of study, multiple sources of data or subgroups of participants), thick description of the study setting and participants to enhance transferability, including multiple quotes (data) to support study findings, maintaining an audit trail with copies of all study‐related material (including raw material and analytic products for each stage of the study) and following the consolidated criteria for reporting qualitative studies (COREQ) [25] accompanied by a completed checklist (see Appendix S1).

### 2.7 Ethical Approval

The study received ethical approval from Makerere University School of Biomedical Sciences Research and Ethics Committee (approval number: SBS‐2021‐24) and the Uganda National Council of Science and Technology (approval number: HS1889ES). Further affirmation of ethical clearance was granted by the Queen's University Belfast Faculty of Medicine, Health and Life Sciences Research Ethics Committee (approval number: MHLS 21_135).

## 3 Results

### 3.1 Characteristics of the Study Participants

Eleven out of the 21 people initially contacted participated in a key informant interview (response rate 52.4%). Six (54.5%) participants had a medical background (i.e., primarily trained as medical doctors) (Table 2). Most stakeholders were male (91%) and practicing or working in a public institution (54.4%).

**TABLE 2:** Characteristics of stakeholders included in the study.

Stakeholder characteristics	*n*	%
Sex		
Male	10	90.9
Female	1	9.1
Background		
Medical	6	54.5
Non‐medical	5	45.5
Institution		
Public	6	54.5
Private	4	36.4
Both	1	9.1

### 3.2 Themes and Subthemes

**FIGURE 1:**
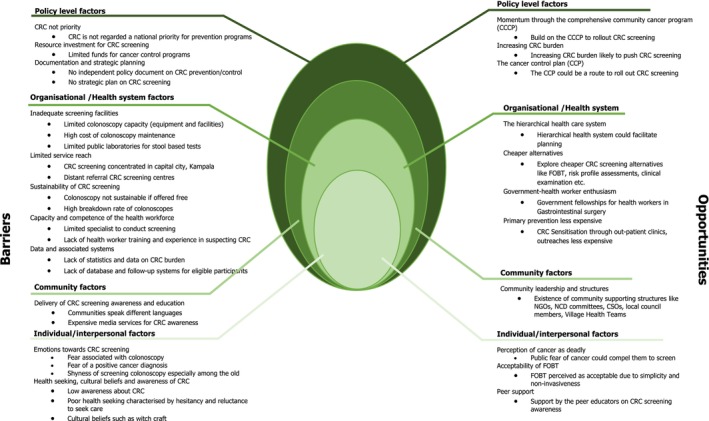
Developing and implementing a CRC screening program in Uganda—stakeholder perceived barriers and opportunities at the different levels of the social ecological model.

Findings were organised into themes based on the four levels of the SEM: policy, organisational/health system, community and individual/interpersonal. Each level included two subthemes: barriers and opportunities (Figure 1).

### 3.3 Theme 1: Policy‐Level Factors

#### 3.3.1 Barriers

##### 3.3.1.1 CRC Not a Current Priority

Stakeholders indicated that the Ugandan government does not recognise CRC as a cancer of national priority, but also noncommunicable diseases (NCDs) more broadly. Stakeholders explained that the government is currently focusing on reducing the burden of infectious diseases. However, with regard to cancer, the focus is on the highest burden cancers, especially cervical, breast and prostate cancers, where some prevention efforts are currently being rolled out (Table 3, Subtheme 1.1a, Quotes 1–2).

‘There are lots of efforts on cervical cancer, there are lots of efforts on breast cancer, there are lots of efforts on prostate cancer. … Issues to do with colorectal cancer, hardly will you hear’ (Non‐medical, Participant 5).

**TABLE 3:** Exemplar quotes from stakeholders on perceived policy‐level factors influencing CRC program development and implementation.

	Exemplar Quote
Theme 1A: Policy‐level barriers	
Subtheme 1.1a: CRC Not a current priority	1. ‘… Its [CRC] burden is increasing but in terms of priority, the highest burden cancers have most of the government priority and even the donor priority in terms of funding for screening and prevention …’ (Non‐medical, Participant 2) 2. ‘There are lots of efforts on cervical cancer, there are lots of efforts on breast cancer, there are lots of efforts on prostate cancer. … Issues to do with colorectal cancer, hardly will you hear’ (Non‐medical, Participant 5)
Subtheme 1.2a: Inadequate resource investment in cancer screening	1. ‘Historically for many years since the UCI [Uganda Cancer Institute] has been in Mulago and even the Mulago hospital itself, prevention aspects of cancer has not been taken seriously in terms of resource allocation and the personnel to do it …’ (Non‐medical, Participant 1) 2. ‘The only thing you will face is just to make sure that the resources for implementation are there. We are talking of implementation, which is just not in Kampala—you know this business of Kampala only has killed us a lot …’ (Non‐medical, Participant 5)
Subtheme 1.3a: Absence of documentation and strategic planning	1. ‘I think the only document I have seen was for cervical cancer. I have not seen any relating to colorectal’ (Medical, Participant 2). 2. ‘As long as I have not seen that plan [strategic plan] yet, and neither has someone reached out to me … I feel that is where we need to start’ (Medical, Participant 1)
Theme 1B: Policy level Opportunities	
Subtheme 1.1b: Momentum through the comprehensive community cancer program	1. ‘We can build on the UCI Comprehensive Community Cancer Program, which is already providing cancer prevention and early detection services to the country’ (Non‐medical, Participant 1)
Subtheme 1.2b: Increasing disease burden	1. ‘… the current shift in the burden from infectious diseases, now we are seeing an increase in the NCD burden—so that also hopefully will shift the donor landscape and we now start thinking of addressing this increasing burden of CRC’ (Non‐medical, Participant 2) 2. ‘… what is emerging is that we are increasingly getting alarmed and alerted by the numbers of colorectal cancers seen in the young, especially males, young adults of between ages 30 to almost 50 years or so … we have started encouraging adults to be screened’ (Medical, Participant 3)
Subtheme 1.3b: The cancer control plan	1. ‘In the draft National Cancer Control Plan that we have been working on, we provided for the development of a screening program for screenable cancers, and I think CRC was included. So that means that if there is going to be development, it is being included’ (Non‐medical, Participant 5) 2. ‘So, what I know is that there have been efforts at the UCI to develop a cancer control program or a cancer control plan. I do not know if that document is already there. I am not sure, but what I know, in the past there was a plan to develop a document of that kind …’ (Medical, Participant 4)

Although some stakeholders highlighted that CRC is starting to gain attention from policy makers and is being discussed to a smaller extent during policy meetings, intentions for a population‐based CRC screening program have not been discussed yet.

##### 3.3.1.2 Resource Investment in the Screening Program

Stakeholders suggested that cancer prevention has historically not been prioritised by the government through the Ministry of Health. Furthermore, cancer services are highly concentrated in the capital, Kampala. Some stakeholders identified these as barriers to accessing screening services (Table 3, Subtheme 1.2a, Quotes 1–2).

‘The only thing you will face is just to make sure that the resources for implementation are there. We are talking of implementation, which is just not in Kampala—you know this business of Kampala only has killed us a lot ….’ (Non‐medical, Participant 5)

##### 3.3.1.3 Documentation and Strategic Planning

A general cancer prevention plan for Uganda exists, and for some cancers, such as cervical cancer, there are disease‐specific strategic plans within the Ministry of Health. However, to date, no such policy documents exist specifically for CRC prevention or control. Stakeholders identified this as a barrier and highlighted the need for such a document to be developed if the government is to show serious intentions of initiating a CRC screening program (Table 3, Subtheme 1.3a, Quote 1).

‘I think the only document I have seen was for cervical cancer. I have not seen any relating to colorectal’ (Medical, Participant 2).

In particular, some medical stakeholders stressed the fact that until the government documents its plans on CRC screening, it will be impossible to start a CRC screening and prevention program in Uganda (Table 3, Subtheme 1.3a, Quote 2).

#### 3.3.2 Opportunities

##### 3.3.2.1 Momentum Through the Comprehensive Community Cancer Program

Stakeholders agreed that the existence of the comprehensive community cancer program at the UCI could push the development and implementation of a CRC screening program. Some stakeholders believed that this is an opportunity for a CRC screening program to be rolled out to the community via existing outreach programs (Table 3, Subtheme 1.1b, Quote 1).

##### 3.3.2.2 Increasing CRC Burden

The increasing burden of CRC within Uganda was identified as a potential opportunity for enhancing CRC prevention and control efforts at both a policy and funding level. The increase in CRC, particularly among young males, is a compelling reason for health workers to encourage the public to get screened. Some stakeholders also anticipate a shift in donor funding patterns to match the epidemiological shift from infectious diseases to NCDs (Table 3, Subtheme 1.2b, Quotes 1–2).

‘… what is emerging is that we are increasingly getting alarmed and alerted by the numbers of colorectal cancers seen in the young, especially males, young adults of between ages 30 to almost 50 years or so … we have started encouraging adults to be screened’ (Medical, Participant 3).

##### 3.3.2.3 The Uganda Cancer Control Plan (CCP)

Although stakeholders referred to the development of a general CCP for Uganda, there were mixed perceptions of its current status. Nonetheless, they shared a common belief that such a document had been or was being developed and that screening for CRC had been mentioned. Stakeholders explained that the CCP would provide strategies for addressing the growing cancer burden, including the development of a detection program for screenable cancers that include CRC (Table 3, Subtheme 1.3b, Quotes 1–2).

### 3.4 Theme 2: Health System/Organisational Level Factors

#### 3.4.1 Barriers

##### 3.4.1.1 Limited Screening Facilities

A frequently identified issue was the limited capacity for CRC screening due to limited equipment and facilities in Uganda. Specifically, stakeholders asserted that the country has few screening centres and colonoscopy equipment.

‘… and then, of course availability of the screening facilities as well. I think there is a limited availability of endoscopies generally in the country’ (Medical, Participant 2).

Some medical stakeholders expressed concerns regarding the extra cost associated with colonoscopy such as equipment maintenance, infection prevention and medicines for bowel preparation prior to endoscopy (Table 4, Subtheme 2.1a, Quotes 2–3). Reflecting on the cost, a few stakeholders highlighted the use of second‐hand colonoscopy machines donated from European countries (Table 4, Subtheme 2.1a, Quote 4).

‘Even the scopes I started training on with were donations and rejects from Europe, from Italy actually …’ (Medical, Participant 5).

**TABLE 4:** Exemplar quotes from stakeholders on perceived health system‐level factors influencing CRC program development and implementation in Uganda.

	Exemplar quotes
Theme 2A: Health system barriers	
Subtheme 2.1a: Limited screening facilities	1. ‘… and then, of course availability of the screening facilities as well. I think there is a limited availability of endoscopies generally in the country’ (Medical, Participant 2) 2. ‘So the challenge with those tests [colonoscopy], what makes them almost inaccessible, is the associated costs’ (Medical, Participant 1) 3. ‘So one of the biggest challenges you would have if you were to screen using colonoscopy, it would be the cost …’ (Medical, Participant 2) 4. ‘Even the scopes I started training on with were donations and rejects from Europe, from Italy actually …’ (Medical, Participant 5) 5. ‘… in our health care settings, some facilities have the equipment for CRC screening other than via the FOBT but some small thing needed for screening, like the reagent, isn't there so the equipment can't be used’ (Non‐medical, Participant 1)
Subtheme 2.2a: Limited service reach	1. ‘So when you look at for example colonoscopy in Kampala, if you are to say government versus private, “who is providing more screening?” actually it is private’ (Medical, Participant 1) 2. ‘Most of the screening really happens at the Uganda Cancer Institute [UCI] for those who are able to reach the UCI’ (Non‐medical, Participant 5) 3. ‘We need to set up multiple centres where [you know] these services can be provided so that not everybody has to come to the national referral hospital because most of these diseases [you know] they are not for the urban people. Even people in the rural areas need these services’ (Medical, Participant 4)
Subtheme 2.3a: Sustainability of screening services	1. ‘Now, there is a lot of wear and tear in these machines because of manipulation. You can use that scope and if, for example, you have different skillsets of people, one person can use it and spoil it …’ (Medical, Participant 4) 2. ‘The moment you say, “there is a free colonoscopy centre,” within two months it will be broken down and not working. Everything is high maintenance; I cannot fully explain it for you’ (Medical, Participant 1) 3. ‘In the long run, I don't think it [colonoscopy] is sustainable. I think the best thing would be to allow for it to be subsidised to a minimal cost such that whoever is getting this service pays something’ (Medical, Participant 4)
Subtheme 2.4a: Capacity and competence of the health workforce	1. ‘If you were to do a nation‐wide coverage, the specialists are also few. You will need to train more of them to provide a complete countrywide coverage’ (Medical, Participant 2) 2. ‘… colonoscopy services are not readily available. Even if they are available, personnel with skills of doing a good colonoscopy, they are still very few in the country’ (Medical, Participant 5) 3. ‘The other thing is that, the ignorance stretches to our clinicians. Clinicians may take long to suspect cancer of the colon. … people end up being diagnosed when it is late’ (Medical, Participant 3) 4. ‘It begins with our health providers, “Do they have an experience?” Not many primary health providers have that experience in having a high index of suspicion of such a problem [colorectal cancer]’ (Medical, Participant 4)
Subtheme 2.5a: Data and associated systems	‘Even where there have been cases of CRC, there is no adequate information, documentation, and data to be relied on. So I think that's why the government may not be funding this …’ (Non‐medical, Participant 3) ‘We need a survey on the problem itself. Just to understand, “what studies have been done to determine the ages, the common ages of presentations, the different [CRC] types, the molecular classifications of the cancers?” I think that baseline information needs to be ready’ (Medical, Participant 4) 3 ‘… the database of eligible people is not yet well set up in our country, even for all other cancer services. You cannot think of having a good database only for cancer when there are other disease that also have the same angle. We would be too aggressive as the cancer control people’ (Non‐medical, Participant 1) 4 ‘… using the cervical cancer experience, one of the challenges we faced was referral—following up positive cases and putting in place a patient monitoring system’ (Non‐medical, Participant 2)
Theme 2B: Health system opportunities	
Subtheme 2.1b: The hierarchical health care system	‘… the network of the health care system in the country is well organised and the health service delivery is well coordinated. We at least know that there is health centre III, there is health centre IV and there is a general hospital and of course the regional referral hospital. That means that for any service you want, you will definitely be able to predict which health service level can provide it’ (Non‐medical, Participant 3) ‘… the referral hospitals is where you find consultants; that would be the best [to start from]’ (Medical, Participant 5) ‘… we hope to use the regional referral hospitals and any regional cancer centres that UCI will have come up with’ (Medical, Participant 3) ‘I think for starters, I would say, “start one screening program at the UCI, and then we roll it out elsewhere”’ (Medical, Participant 6)
Subtheme 2.2b: Cheaper CRC screening alternatives	‘… for FOBT, any laboratory, any health centre IV laboratory should be able to do it. That is why I told you that the beauty with FOBT, it is scalable and it is very cheap. For mass screening, it is more applicable …’ (Medical, Participant 1) ‘Well‐designed screening programs of course should focus on patients at high risk, with positive family history, probably with other risk factors i.e. smoking, alcohol, and then faecal occult blood test and then the patient ends up with a colonoscopy’ (Medical, Participant 5) ‘We can do a clinical examination that involves looking at them [the patients], looking at the abdomen, doing a digital rectal examination. Depending on what we find, we can request all these guiding screening tests especially patients who are above [for example] 50 years, because we know that the risk of this cancer increases from like the 5th decade’ (Medical, Participant 4)
Subtheme 2.3b: Government—Health workers enthusiasm	‘I think about 50 doctors were sent on government money to go to India to train, not in colorectal cancer, but in surgery, advanced surgical techniques. So from that point of view I think government did well’ (Medical, Participant 1) ‘Even currently, people went and trained in India and they came back. They are colon surgeons who went to train specifically …’ (Medical, Participant 5) ‘Most of us trained ourselves, you get your money and you train’ (Medical, Participant 1)
Subtheme 2.4b: Primary prevention less expensive	‘Prevention as primary prevention will have fewer issues compared to resource issues that must be addressed to ensure the intervention [CRC screening] is implemented’ (Non‐medical, Participant 1) ‘Yes, in our outreach programs we sensitise people. When speaking about cancer risk factors, we [also] address risk factors for all the most common cancers, including CRC, because CRC always shares some of the risk factors with other types of cancer’ (Non‐medical, Participant 1) ‘… when we are giving a session on health education, like, as you well know that the predisposing factors for most of the cancers are almost the same i.e. smoking, alcohol, age related factors, infections, heredity … those ones. When we are educating the public about cancers, we mention those different factors, which can lead to cancer including that of the colon and rectum’ (Non‐medical, Participant 4)

Public laboratories for conducting stool‐based CRC tests, such as the faecal occult blood test (FOBT), were also reported to be insufficient, which could act as a barrier if a nationwide FOBT program were to be considered. Stakeholders also explained that most public hospital laboratories have difficulty maintaining an adequate stock of reagents to conduct certain tests (Table 4, Subtheme 2.1a, Quote 5).

##### 3.4.1.2 Limited Service Reach

Stakeholders confirmed that existing CRC screening services are mostly offered by private facilities and laboratories. Only the national referral hospital in Kampala and a few regional referral hospitals offer this service (Table 4, Subtheme 2.2a, Quotes 1–2).

‘So, when you look at for example colonoscopy in Kampala, if you are to say government versus private, “who is providing more screening?” actually it is private’ (Medical, Participant 1).

To address this challenge, stakeholders identified the need for more screening centres across the country to ensure equity in service provision between the rural and urban populations (Table 4, Subtheme 2.2a, Quote 3).

##### 3.4.1.3 Sustainability of Screening Services

Some stakeholders, especially those from medical specialties, questioned the sustainability of offering free‐of‐charge CRC screening services, in particular colonoscopy. Due to the anticipated high rate of machine breakdown with increasing use and varying specialist skillsets, a user fee or subsidised cost was identified by stakeholders as being necessary (Table 4, Subtheme 2.3a, Quotes 1–3).

‘The moment you say, “there is a free colonoscopy centre,” within two months it will be broken down and not working. Everything is high maintenance; I cannot fully explain it for you’ (Medical, Participant 1).

##### 3.4.1.4 Capacity and Competence of the Health Workforce

Current workforce capacity and skillsets were also highlighted as potential barriers to initiating a CRC screening program. The stakeholders indicated that there are not enough specialists, such as colonoscopists, anaesthesiologists, pathologists and gastrointestinal surgeons in Uganda to oversee an organised CRC screening program. Such a program, in fact, would place an increased workload on existing staff. The lack of adequate numbers of support health workers, such as nurses needed to support the successful implementation of a screening program, was also mentioned as a barrier (Table 4, Subtheme 2.4a, Quotes 1–2).

Stakeholders also mentioned that most healthcare professionals in Uganda do not have adequate knowledge, training and experience in diagnosing and managing CRC (Table 4, Subtheme 2.4a, Quotes 3–4).

‘It begins with our health providers, “Do they have an experience?” Not many primary health providers have that experience in having a high index of suspicion of such a problem [colorectal cancer]’ (Medical, Participant 4).

##### 3.4.1.5 Data and Associated Systems

The lack of local robust evidence characterising CRC burden in Uganda was identified as another barrier to developing evidence‐based CRC screening policies and programs. Stakeholders felt concerned that unless such data became available, the government would not be compelled to fund a CRC screening program (Table 4, Subtheme 2.5a, Quotes 1–2). Some stakeholders were in favour of conducting an initial feasibility study to acquire data to inform a screening program. This would include evidence of public willingness to pay for the screening services and a more complete profile of CRC burden within Uganda.

The absence of a database of CRC screening eligible participants and the lack of systems to follow up on suspected or diagnosed cases of CRC were also identified as barriers (Table 4, Subtheme 2.5a, Quotes 3–4).

‘… the database of eligible people is not yet well set up in our country, even for all other cancer services. You cannot think of having a good database only for cancer when there are other disease that also have the same angle. We would be too aggressive as the cancer control people’ (Non‐medical, Participant 1).

#### 3.4.2 Opportunities

##### 3.4.2.1 The Hierarchical Health Care System

The hierarchical nature of Uganda's health system could potentially support the planning and integration of a CRC screening program. Each level of the system has clearly defined roles in terms of service delivery, and this could help identify which health level is most suited for providing CRC screening services (Table 4, Subtheme 2.1b, Quote 1).

Some stakeholders also believed that a top‐down approach that would first provide screening services at the regional referral hospitals, planned cancer centres or the UCI would be the most suitable strategy. This would allow the new program to benefit from existing infrastructure and specialists, thereby reducing the need for additional resources and qualified personnel at lower‐level health facilities (Table 4, Subtheme 2.1b, Quotes 2–4).

‘I think for starters, I would say, “start one screening program at the UCI [Uganda Cancer Institute], and then we roll it out elsewhere”’ (Medical, Participant 6).

##### 3.4.2.2 Cheaper CRC Screening Alternatives

Medical specialists identified the presence of cheap, scalable and readily available screening alternatives for Uganda. These include FOBT, clinical examinations, family history and risk factor profiles. Using a combination of such screening methods initially could help reduce the burden on colonoscopy services, especially if screening focused on population subgroups identified as being at higher risk of CRC. In addition, some stakeholders mentioned conducting a digital rectal exam for early detection of CRC for patients who present with symptoms (Table 4, Subtheme 2.2b, Quotes 1–3).

‘… for FOBT, any laboratory, any health centre IV laboratory should be able to do it. That is why I told you that the beauty with FOBT, it is scalable and it is very cheap. For mass screening, it is more applicable …’ (Medical, Participant 1).

##### 3.4.2.3 Government—Health Workers Enthusiasm

Stakeholders with a medical background acknowledged positive action by the government to support health workers through providing training fellowships for international training in gastrointestinal surgery and advanced surgical techniques. These skilled individuals could greatly enhance a CRC screening program (Table 4, Subtheme 2.3b, Quotes 1–2).

‘I think about 50 doctors were sent on government money to go to India to train, not in colorectal cancer, but in surgery, advanced surgical techniques. So, from that point of view, I think government did well’ (Medical, Participant 1).

In addition, one medical stakeholder commended the enthusiasm of local health workers who paid for their own training (Table 4, Subtheme 2.3b, Quote 3).

##### 3.4.2.4 Primary Prevention Less Expensive

Stakeholders indicated that the current financial situation of the country, coupled with the high infectious disease burden and the focus on high‐burden cancers, might limit the feasibility of developing and implementing a CRC screening program at present. Instead, many stakeholders indicated that a primary CRC prevention program would be considerably more feasible and less resource intensive, especially for a low‐income country like Uganda (Table 4, Subtheme 2.4b, Quote 1).

‘Prevention as primary prevention will have fewer issues compared to resource issues that must be addressed to ensure the intervention [CRC screening] is implemented’ (Non‐medical, Participant 1).

Stakeholders expressed various ways in which they currently conduct primary prevention of CRC in Uganda indirectly, such as incorporating awareness messages of CRC and its risk factors into existing community outreach programs, outpatient clinic days and media platforms used to sensitise the public to cancer in general (Table 4, Subtheme 2.4b, Quotes 2–3).

### 3.5 Theme 3: Community‐Level Factors

#### 3.5.1 Barriers

##### 3.5.1.1 Delivery of CRC Awareness and Education

Some stakeholders identified language as a potential challenge for the delivery of educational messages on CRC and screening. As communities within different regions of Uganda speak and understand various languages (over 40 languages are spoken in total in Uganda), translating materials into different languages using local translators or bilingual experts would add additional cost and require additional resources (Table 5, Subtheme 3.1a, Quote 1).

‘… of course, the languages in our population are varied. If I have to go to the communities, you have to translate a lot of information to so many languages. For that matter therefore, we need a lot of resources to change the information into different languages’ (Non‐medical, Participant 3).

**TABLE 5:** Exemplar quotes from stakeholders on perceived community‐level factors influencing CRC program development and implementation in Uganda.

	Exemplar Quote
Theme 3A: Community‐level barriers	
Subtheme 3.1a: Delivery of CRC awareness and education	‘… of course, the languages in our population are varied. If I have to go to the communities, you have to translate a lot of information to so many languages. For that matter therefore, we need a lot of resources to change the information into different languages’ (Non‐medical, Participant 3) 2 ‘… of course the other challenge is dealing with the media. The media here is very expensive and profit driven’ (Non‐medical, Participant 3)
Theme 3B: Community level opportunities	
Subtheme 3.1b: Community leadership and structures	‘We have the support from the leaders. We have structures in terms of the NCDs council. There is a committee, which heads the running of NCDs in the district with committee members, chairperson and the like. Then, the other leaders—the political, technical leaders we have their support …’ (Non‐medical, Participant 4) ‘Of course for us as a CSO, one thing that we have done, we have been able to mobilise the media on NCDs and cancer specifically …’ (Non‐medical, Participant 3)

Although media services have the potential to promote CRC screening awareness to a wider audience, one stakeholder highlighted the expensive nature of media services within Uganda as a potential barrier to their use in such a program (Table 5, Subtheme 3.1a, Quote 2).

#### 3.5.2 Opportunities

##### 3.5.2.1 Community Leadership and Structures

Several stakeholders recognised the important roles that local community leaders fill regarding public health initiatives such as a CRC screening program. These include local council chairpersons, staff and volunteers in community non‐governmental organisations/civil society organisations and village health team members. Such community leaders could help create awareness about screening services, mobilise and encourage people within the target age group to go for screening and timely follow‐up of screen‐positive patients for treatment (Table 5, Subtheme 3.1b, Quotes 1–2).

‘We have the support from the leaders. We have structures in terms of the NCDs council. There is a committee, which heads the running of NCDs in the district with committee members, chairperson and the like. Then, the other leaders—the political, technical leaders we have their support …’ (Non‐medical, Participant 4).

### 3.6 Theme 4: Interpersonal/Individual Factors

#### 3.6.1 Barriers

##### 3.6.1.1 Emotions Towards CRC Screening

A common view held by stakeholders was that most people would be afraid to have a colonoscopy, especially those who are asymptomatic or perceive themselves to be healthy. Stakeholders asserted that this could be due to the invasiveness and ‘theatre kind of’ set‐up associated with the colonoscopy procedure (Table 6, Subtheme 4.1a, Quotes 1–3).

‘… there are people who are symptomatic, those ones are usually more likely to accept the [colonoscopy] procedure—actually, they come demanding for it. “I saw blood in my stool—can you please check and see?” Now for the people who are “healthy”, it is not acceptable in our population’ (Medical, Participant 1)

**TABLE 6:** Exemplar quotes from stakeholders on perceived individual/interpersonal factors influencing CRC program development and implementation in Uganda.

	Exemplar Quote
Theme 4A: Interpersonal and individual level barriers	
Subtheme 4.1a: Emotions towards CRC screening	‘… there are people who are symptomatic, those ones are usually more likely to accept the [colonoscopy] procedure—actually, they come demanding for it. “I saw blood in my stool—can you please check and see?” Now for the people who are ‘healthy’, it is not acceptable in our population’ (Medical, Participant 1) ‘Let us do some good background work to make sure that people find it [colonoscopy] more acceptable. Because I see anything that involves theatre kind of set up, people are not always eager to go’ (Medical, Participant 2) ‘The one [screening test] that might have a challenge is the colonoscopy; because it is invasive’ (Non‐medical, Participant 2) 4 ‘… when you talk about cancer in general, people tell themselves, “if you tell me I have cancer—it is like a death sentence.” … Someone may think that they are better off not knowing after all, there is nothing much you are going to do about it’ (Medical, Participant 2) 5 ‘… the population would most likely not respond positively. In most cases, when someone is asymptomatic, he/she does not see why they should go and subject him/herself. … people do not want to get bad news because they are still enjoying themselves’ (Medical, Participant 3) 6 ‘Some few members of the public feel shy—especially we have seen with cancer of the cervix where people have to expose themselves, the lithotomy positions, especially the older ages find they are three times older than the provider is. … They do not feel comfortable’ (Non‐medical, Participant 4)
Subtheme 4.2a: Health seeking, cultural beliefs and awareness about CRC	‘First of all, there is low knowledge about colorectal cancer which we have seen—and generally cancer at large …’ (Non‐medical, Participant 2) ‘… you will find somebody who is educated, they have had bleeding in the rectum and, for example, they are busy at work. They cannot take time off to go and check themselves. First of all, they do not have information that it is a serious problem’ (Medical, Participant 4) ‘I think we live in a community generally where people would not want to go to a hospital when they feel well. So the screening per se, the uptake may not be fine even beyond colorectal. Because the health seeking behaviour is usually related directly to feeling of poor health’ (Medical, Participant 2) 4 ‘Some community members are so much traditional that they attribute some conditions like cancers to witchcraft. … Some people will take time to seek health care services—they start with prayers, they start with traditional healers, they go to these herbalists, something of that kind. By the time they come to our facilities, it is too late’ (Non‐medical, Participant 4)
Subtheme 4.3a: Financial constraints	‘There are other social issues that come into play; like things to do with money, transport and the like …’ (Medical, Participant 5) ‘… because of the cost, there are people who would actually want to have the test but they cannot afford, so that can be a limitation in one way or the other’ (Medical, Participant 1)
Theme 4B: Interpersonal and individual level opportunities	
Subtheme 4.1b: Perception of cancer as deadly	‘People are aware that cancer is a problem … majority of people now think that the cause of death for several people is possibly cancer’ (Non‐medical, Participant 1) ‘Without looking at the types of cancer, but the moment you mention the word “cancer” generally, people can open their ears to listen. So I think that is where we can start from …’ (Non‐medical, Participant 3)
Subtheme 4.2b: Likely acceptability of FOBT	‘I think it [FOBT] can be acceptable because for it all what the patient does is provide a stool sample. An FOBT can work very well in the community’ (Medical, Participant 4) ‘I think it should be about educating the public. I mean, “If you teach me and you tell me the FOBT is of much benefit” then I think people would try to accept the test’ (Medical, Participant 6) ‘Because the FOBT is not invasive, it is generally more acceptable …’ (Medical, Participant 1)
Subtheme 4.3b: Peer support	‘… we have what we call “peer educators”. These are people who are living with the condition and they have been trained to do counselling to their fellow members, identify members who need psychosocial support etc …. So when we start [the screening program], we can select peer members from those who are attending the service’ (Non‐medical, Participant 4)

Stakeholders further added that there is a general fear of a positive cancer diagnosis among the public, which may negatively influence participation in a cancer screening program. Cancer may be perceived as a death sentence, and most people, particularly those who are well, may be hesitant to participate, perhaps believing that nothing can be done should they receive a positive diagnosis (Table 6, Subtheme 4.1a, Quotes 4–5).

Basing their views on experiences drawn from cervical cancer screening, one stakeholder mentioned that shyness/embarrassment may also act as a barrier to screening participation involving colonoscopy (Table 6, Subtheme 4.1a, Quote 6).

‘Some few members of the public feel shy—especially we have seen with cancer of the cervix where people have to expose themselves, the lithotomy positions, especially the older ages find they are three times older than the provider is. … They do not feel comfortable’ (Non‐medical, Participant 4).

##### 3.6.1.2 Health‐Seeking, Cultural Beliefs and Awareness About CRC

A common view held by stakeholders was that public awareness and knowledge of CRC are limited, and they believe this could hinder screening uptake. For some, limited knowledge may delay or increase reluctance to seek health care even when symptomatic (Table 6, Subtheme 4.2a, Quotes 1–3).

‘… you will find somebody who is educated, they have had bleeding in the rectum, and for example, they are busy at work. They cannot take time off to go and check themselves. First of all, they do not have information that it is a serious problem’ (Medical, Participant 4).

Also, partly due to the poor awareness about CRC risk factors, stakeholders widely believed that some people still believe that diseases such as CRC are caused by witchcraft and so would seek help from non‐medical practitioners. This could delay presentation for screening or early diagnosis (Table 6, Subtheme 4.2a, Quote 4).

##### 3.6.1.3 Financial Constraints

Stakeholders recognised that money was likely to play a contributing role in the public's decision on whether to participate in cancer screening. In particular, costs associated with transport to screening centres were identified as a potential barrier to participation. Furthermore, unless provided free of charge, having to pay for the screening service could act as a further barrier (Table 6, Subtheme 4.3a, Quotes 1–2).

‘There are other social issues that come into play; like things to do with money, transport and the like …’ (Medical, Participant 5).

#### 3.6.2 Opportunities

##### 3.6.2.1 Perception of Cancer as Fatal

Although fear associated with receiving a cancer diagnosis was identified as a potential barrier to screening, some stakeholders believed that the perception of cancer as a deadly disease and increasing awareness within the community might actually have the opposite effect and encourage people to take steps for prevention and early detection (Table 6, Subtheme 4.1b, Quotes 1–2).

‘Without looking at the types of cancer, but the moment you mention the word “cancer” generally, people can open their ears to listen. So I think that is where we can start from …’ (Non‐medical, Participant 3).

##### 3.6.2.2 Likelihood of FOBT Acceptance

The majority of stakeholders believed the public would find FOBT to be acceptable due to its simplicity and non‐invasive nature. Stakeholders also believed that the uptake of FOBT would be good, provided awareness was first created about its benefits (Table 6, Subtheme 4.2b, Quotes 1–3).

‘I think it [FOBT] can be acceptable because for it all what the patient does is provide a stool sample. An FOBT can work very well in the community’ (Medical, Participant 4).

##### 3.6.2.3 Peer Support

Some stakeholders, particularly those from non‐medical backgrounds, believed that a screening program would benefit from peer educators who could provide support for patient follow‐up and psychosocial counselling and services. This cadre could include people who have the lived experience of being diagnosed with CRC. They could also advocate for CRC awareness campaigns to encourage others to present for CRC screening or be diagnosed early (Table 6, Subtheme 4.4b, Quote 1).

## 4 Discussion

This study explored stakeholders' perceived barriers and opportunities related to developing and implementing a CRC screening program in Uganda. At the policy level, some of the barriers cited in this study (e.g., lack of prioritisation of CRC, limited financing or resources) have been previously documented in other LMICs, including Ghana [26], Egypt [21] and Mexico [14]. In the Ghanaian study, stakeholders mentioned that CRC is not a national priority and has not been incorporated into their national health insurance scheme despite the government endorsing national screening guidelines in 2011 [26]. Similar to our study, these findings most likely reflect the low CRC incidence rates (i.e., 4.7 per 100,000 in Ghana, and 7.3 per 100,000 in Uganda [1]), with policy makers not yet convinced that CRC is a major problem. This is also a consideration in light of the much higher rates of breast cancer (23.3 per 100,000) and cervical cancer (53.8 per 100,000) in Uganda, which are among the top three most incident cancers [1]. Interestingly, the rising CRC burden within Uganda was identified as an opportunity within the current study, which stakeholders felt could compel the government to start discussions for a national screening program.

Ngan et al. estimated that the cost of a home‐based CRC screening program among 50–75 year‐olds in Malaysia was about 74.6 million international dollars in the first year of program initiation [27]. In Uganda, the health sector budget of 2022/2023 was approximately 2.2 trillion Ugandan shillings (≈595,000,000 USD) [28, 29], with 17% (≈101,150,000 USD) allocated to non‐communicable diseases [30]. Introducing a CRC screening program is likely to be a resource‐draining initiative unless extra funding is obtained from other sources or through additional budget adjustments. Participants in this study indicated that implementing primary prevention measures (e.g., CRC awareness creation and mass education campaigns) and using a top‐down approach to offer CRC screening (i.e., starting in the higher‐level facilities, using less costly methods for primary screening, such as FOBT, CRC risk profile assessments, clinical examinations) could help overcome financial and resource requirements.

Unlike other LMICs that have examined barriers and opportunities for implementing CRC screening programs where guidelines are already in place or pilot projects have been conducted [8, 26, 31, 32], Uganda currently lacks this information. Therefore, it is crucial to use locally generated recommendations when developing a CRC screening program, considering the contextual differences.

Most barriers cited at the health system level in this study (e.g., limited screening facilities, insufficient health workforce, lack of health worker training and knowledge on CRC, lack of epidemiological CRC data, absence of follow‐up mechanisms and database systems) have been reported elsewhere, for example, in Egypt [21], Mexico [14], Morocco [8] and Nigeria [33]. Besides, the limited capacity for screening and capacity for the management and treatment of those diagnosed are also insufficient in many LMICs [34], hence pointing back to the resource gap identified at the policy level. To address shortfalls in endoscopy service provision, some HICs have trained nurse endocopists [35]. Despite not being identified by stakeholders in the current study, this area warrants further assessment for feasibility if a CRC screening program were to be initiated in the country.

Some of the unique health system barriers mentioned by stakeholders in our study that we did not find reported elsewhere include concerns regarding service reach or coverage of CRC screening services and sustainability of free colonoscopy services. Stakeholders emphasised that currently CRC screening services are offered at the main public tertiary hospital in Kampala and in a few private hospitals/laboratories within town centres. This can create disparities in the health of the population due to inequitable access to CRC screening services, although limited documentation exists reporting such disparities. Furthermore, limited CRC service provision across Uganda may lead to inaccurate statistics in terms of CRC burden. Drawing on successful cervical cancer screening programs, where screening has been integrated into existing reproductive health services [36], future CRC screening programs could benefit from similar arrangements to improve accessibility to and expansion of CRC screening services. For instance, CRC screening could be integrated within existing services such as routine NCD weekly clinic schedules at lower‐level facilities. However, lower‐level facilities would need to be given the necessary equipment (e.g., stool‐based test kits like FOBT) and training for healthcare workers to do the screening. Moreover, some studies in Africa have recommended putting measures in place to retain trained endoscopists in public facilities, as these tend to shift to private facilities, creating a vacuum in service provision in the former [37].

With regard to concerns over colonoscopy sustainability, stakeholders regarded this procedure as a high‐maintenance service that requires special care of equipment (e.g., extra cleaning for infection control and good handling to prevent high breakdown rate). Issues related to the maintenance of colonoscopy machines have also been reported in Nigeria, where continuous provision of colonoscopy services could not be maintained due to machine breakdown [38]. To meet colonoscopy‐related maintenance costs, stakeholders mentioned that a subsidised user fee could be imposed on service users. However, this could exacerbate access issues for lower socio‐economic groups, as shown in other settings [39]. Further research is needed to assess the feasibility of a subsidised fee model for CRC screening before a national program can be rolled out.

At the community level, stakeholders identified that the existence of multiple languages in Uganda may make it difficult to mount a national or even regional program of public education and awareness about CRC and screening. Furthermore, media services to promote these messages are expensive. The latter problem could be minimised through government partnerships with key media outlets. Moreover, the government may also need to take advantage of the existing community structures that stakeholders identified as opportunities to support the screening program, such as the local council chairpersons, NCD committees, local NGO/CSOs and the village health team members. Stakeholders assumed that such leaders are conversant with the specific languages within their regions and can create and provide support mechanisms for the screening program. Indeed, in a 2021 systematic review that included studies from the USA, Asia and Africa, community health workers were found to offer education (*n* = 30 studies), screening support (*n* = 7 studies) and navigation (*n* = 6 studies) to improve screening rates (*n* = 23 studies) of breast cancer [40]. The ability of community leaders and health workers to share information with their wider community may help to address the individual and interpersonal barriers identified in this study, such as poor health‐seeking habits, fear of the screening test, cultural beliefs regarding cancer, such as ‘witchcraft being regarded as a cause of cancer’ and low CRC awareness, among others. For instance, in the Carolinas Cancer Education and Screening (CARES) project in the United States, the involvement of community volunteers was reported to positively influence community beliefs and attitudes towards screening and being screened for CRC [41].

### 4.1 Study Strengths and Limitations

This study is the first to explore the perceived barriers and opportunities regarding the development and implementation of a CRC screening program in Uganda according to various specialists who could potentially help develop a national/community‐based CRC screening program. Accepted methodological guidelines were followed when designing and conducting the study, including the analysis and interpretation of the data.

However, this study did have some limitations. Few community, interpersonal and individual‐level barriers and opportunities were identified, which may reflect the stakeholders' professional backgrounds being mainly policy, program and screening experts with no inclusion of patients or community members who could perhaps have provided in‐depth insights on such factors. Future studies should seek to explore patient barriers. Extensive efforts were made to identify key personnel in cancer programming, policy and screening in Uganda. However, as cancer service provision has not been mapped across the country, it is possible that some individuals were not identified through the scoping exercise. In addition, the transferability of these findings may only be relevant to LMICs without an existing CRC screening intervention, program or policy in place. Countries with existing CRC screening interventions or programs have an existing backbone and government will for such programs, and screening guidelines are likely to be already in place.

## 5 Conclusion

This study has identified several stakeholder‐perceived barriers and opportunities at all levels of the SEM that could hinder or facilitate the introduction of a successful CRC screening program in Uganda. In general, stakeholders believed that although there are some opportunities that could facilitate implementing such a program, substantial work remains, especially at the policy and health system levels. To advance this goal, it is essential that the government identifies CRC as a national health priority, develops a strategic plan and sets aside adequate resources for a population‐based CRC screening program. Until then, virtually all stakeholders interviewed suggested that the focus of CRC reduction should be on primary prevention through CRC awareness campaigns and mass education. Stakeholders also noted that these interventions are less expensive than screening and yet could be highly beneficial to CRC control in a resource‐constrained setting.

## Author Contributions

**Nicholas Matovu:** conceptualization (equal), formal analysis (equal), investigation (lead), writing – original draft (lead), writing – review and editing (equal). **Helen G. Coleman:** conceptualization (equal), funding acquisition (lead), supervision (lead), writing – review and editing (equal). **Gerald Mutungi:** conceptualization (equal), project administration (equal), writing – review and editing (equal). **Michael Donnelly:** conceptualization (equal), writing – review and editing (equal). **Lynne Lohfeld:** formal analysis (equal), methodology (equal), writing – review and editing (equal). **Brian T. Johnston:** conceptualization (equal), writing – review and editing (equal). **Maurice B. Loughrey:** conceptualization (equal), writing – review and editing (equal). **Noleb M. Mugisha:** conceptualization (equal), supervision (equal), writing – review and editing (equal). **Charlene M. McShane:** conceptualization (equal), formal analysis (equal), funding acquisition (lead), supervision (lead), writing – review and editing (equal).

## Ethics Statement

The study received ethical approval from Makerere University School of Biomedical Sciences Research and Ethics Committee (approval number: SBS‐2021‐24) and the Uganda National Council of Science and Technology (approval number: HS1889ES). Further affirmation of ethical clearance was granted by the Queen's University Belfast Faculty of Medicine, Health and Life Sciences Research Ethics Committee (spproval number: MHLS 21_135).

## Consent

Written informed consent was obtained from every participant prior to enrolment into the study.

## Conflicts of Interest

The authors declare no conflicts of interest.

## Supporting information


Data S1.

## Data Availability

All data relevant for this study are available within the manuscript or as Supporting Information uploaded as part of this paper.
