# Supplementary material for: Developing and Implementing a Colorectal Cancer Screening Program in Uganda: Stakeholder Perceived Barriers and Opportunities
Source: Cancer Med. 2025 Mar 13;14(6):e70662. doi: 10.1002/cam4.70662 (PMC11904429; doi:10.1002/cam4.70662)
Supplement: Supplementary file 1 — Data S1. [file CAM4-14-e70662-s001.docx]

**Appendix 1:** The Interview Guide

**KEY INFORMANT INTERVIEW GUIDE (Institutional stakeholders)**

This interview guide will be used to guide the collection of information from key institutional stakeholders about colorectal cancer (CRC) programming, screening or prevention and control.

**Identification of key informant**

District: ……………………………..

Position: ………………………………

Health facility/ institutional name: …………………………………………

**Questions**

**About the participant**

1. Please can you tell me about yourself?
2. What are your responsibilities in this facility or institution/ what do you do?

*(Probe for: position/delegated tasks, work experience especially in cancer/CRC programing, screening or prevention and control, how long they’ve worked in the facility/institution.)*

1. What are the common malignancies/cancers that are prioritized in terms of prevention and control interventions and screening at the [National/community/health facility level]

*(Probe for interventions such as mass education campaigns, coverage of: existing screening programs, awareness and sensitization programs.)*

1. What programs/campaigns exist in prevention, control and screening for colorectal cancer *(explain in detail what colorectal cancer is, if requested)*

*(Probe: presence of sensitization/awareness campaigns, mass education campaigns, screening program (what level of health facility is screening done at public and private facilities, is there a national, community or health facility CRC screening program or policy document such as guidelines or strategic plan)*

1. What are the primary and secondary screening methods for CRC [at national, community or health facility]?

*(probe: provide the choice of CRC screening methods i.e iFOBT, gFOBT, colonoscopy, sigmoidoscopy, stool DNA testing, colon capsule endoscopy, CT colonography, Double contrast barium enema)*

Primary methods for CRC screening are those that are used first before any follow up tests (usually quick and simple)

Secondary methods are those that follow or are requested in case of positive or unclear results.

1. Describe your understanding of the CRC screening process in (Uganda, your community, health facility)?

*(Probe for: How are people contacted for screening, what happens when they go to a screening center, how are they followed up, how are results from screening communicated, what happens to those who test positive or negative– how are they treated or followed up, how are records kept?, who interprets results?)*

1. In your opinion, what are the challenges in implementing or staging CRC prevention, control and screening interventions [in Uganda, your community, your health facility]

*(probe if not mentioned: availability of policy documents such as strategic plan/screening guidelines, human resource capacity or number, human resource expertise, availability of screening equipment or reagents, cost of screening – is it out of pocket or covered by health insurance or free, data management and follow up of cases, how are potential people reached for screening, acceptability of the screening tests by the people)*

1. In your opinion, what could be hindering people from accessing, seeking or taking screening tests for CRC?

*(probe if not mentioned: knowledge levels on CRC signs or symptoms, awareness of CRC, knowledge on where to seek screening tests, knowledge on screening methods or their existence, cost, distance to screening centers, attitude towards the screening tests or screening itself)*

1. Are you aware of any plans to introduce a CRC screening program, or increase coverage of existing screening programs?

*(Probe for: presence of draft policy documents where CRC is accommodated, training or fellowships for specialists, ongoing surveys on CRC or its screening, new modalities of reaching/encouraging people to screening – telephone, SMS etc; planned awareness or sensitization campaigns, regional screening centers introduced or planned, subsidizing or proving free CRC screening, procurement of more equipment for wider coverage, etc.)*

1. I am providing a copy of the Bowel CAM tool used in the UK to assess CRC awareness and knowledge. What is your opinion on its suitability of use in our local Ugandan setting? (Probe for: appropriateness of questions, comprehensiveness of the questions; validity of the questions)

**SWOT:**

1. What are the main Strengths of current arrangements for CRC screening?
2. What are the main Weaknesses of current arrangements?
3. What are the main Opportunities of current arrangements?
4. What are the main Threats to current arrangements?

**This is the end. Thank you for your time!**

**Supplementary file:**

**Appendix 2:** Consolidated criteria for reporting qualitative studies (COREQ): 32-item checklist

| No | Item | Guide questions/description | Response/Page item included |
| --- | --- | --- | --- |
| Domain 1: Research team and reflexivity | | |  |
| Personal Characteristics | | |  |
| 1. | Interviewer/facilitator | Which author/s conducted the interview or focus group? | NM, see data collection section |
| 2. | Credentials | What were the researcher's credentials? *E.g. PhD, MD* | PhD candidate |
| 3. | Occupation | What was their occupation at the time of the study? | PhD Candidate/student |
| 4. | Gender | Was the researcher male or female? | Male |
| 5. | Experience and training | What experience or training did the researcher have? | PhD student with further training in qualitative methods |
| Relationship with participants | | |  |
| 6. | Relationship established | Was a relationship established prior to study commencement? | Yes |
| 7. | Participant knowledge of the interviewer | What did the participants know about the researcher? e*.g. personal goals, reasons for doing the research* | None, except previous being collaborators |
| 8. | Interviewer characteristics | What characteristics were reported about the interviewer/facilitator? e.g. *Bias, assumptions, reasons and interests in the research topic* | None |
| Domain 2: study design | | |  |
| Theoretical framework | | |  |
| 9. | Methodological orientation and Theory | What methodological orientation was stated to underpin the study? *e.g. grounded theory, discourse analysis, ethnography, phenomenology, content analysis* | Qualitative description, see Study design, setting and population section |
| Participant selection | | |  |
| 10. | Sampling | How were participants selected? *e.g. purposive, convenience, consecutive, snowball* | Purposive; see participant recruitment and sampling section |
| 11. | Method of approach | How were participants approached? e*.g. face-to-face, telephone, mail, email* | Telephone & email; see data collection section |
| 12. | Sample size | How many participants were in the study? | 11 participants; see data collection section & results section |
| 13. | Non-participation | How many people refused to participate or dropped out? Reasons? | 10 people; see results section  Reason: busy schedule |
| Setting | | |  |
| 14. | Setting of data collection | Where was the data collected? e*.g. home, clinic, workplace* | Both at work places and virtually; see data collection section |
| 15. | Presence of non-participants | Was anyone else present besides the participants and researchers? | None |
| 16. | Description of sample | What are the important characteristics of the sample? *e.g. demographic data, date* | Provided in results section, characteristics of participants |
| Data collection | | |  |
| 17. | Interview guide | Were questions, prompts, guides provided by the authors? Was it pilot tested? | Yes; see data collection section |
| 18. | Repeat interviews | Were repeat interviews carried out? If yes, how many? | No |
| 19. | Audio/visual recording | Did the research use audio or visual recording to collect the data? | Yes; see data collection section |
| 20. | Field notes | Were field notes made during and/or after the interview or focus group? | Yes; see data collection section |
| 21. | Duration | What was the duration of the interviews or focus group? | 20-50 minutes; see data collection section |
| 22. | Data saturation | Was data saturation discussed? | Yes; see data collection section |
| 23. | Transcripts returned | Were transcripts returned to participants for comment and/or correction? | No |
| Domain 3: analysis and findings | | | |
| Data analysis | | | |
| 24. | Number of data coders | How many data coders coded the data? | Two; see data analysis section |
| 25. | Description of the coding tree | Did authors provide a description of the coding tree? | Yes; see data analysis section |
| 26. | Derivation of themes | Were themes identified in advance or derived from the data? | Deductive; see data analysis section |
| 27. | Software | What software, if applicable, was used to manage the data? | Microsoft Excel; see data analysis section |
| 28. | Participant checking | Did participants provide feedback on the findings? | Not yet (planned) |
|  | | | |
| 29. | Quotations presented | Were participant quotations presented to illustrate the themes / findings? Was each quotation identified? e*.g. participant number* | Yes; see data analysis section & results section |
| 30. | Data and findings consistent | Was there consistency between the data presented and the findings? | Yes |
| 31. | Clarity of major themes | Were major themes clearly presented in the findings? | Yes; Figure 1 & results section |
| 32. | Clarity of minor themes | Is there a description of diverse cases or discussion of minor themes? | Yes; Figure 1 & results section |

| Stakeholder characteristics | n | % |
| --- | --- | --- |
| Sex |  |  |
| Male | 9 | 90 |
| Female | 1 | 10 |
| Background |  |  |
| Medical | 10 | 100 |
| Non-medical | 0 | 0 |
| Institution |  |  |
| Public | 5 | 50 |
| Private | 4 | 40 |
| Both | 1 | 10 |
| Specialities |  |  |
| Gastroenterologists | 4 | 40 |
| Gastrointestinal surgeons | 3 | 30 |
| Oncologists | 2 | 20 |
| Medical Officer (District level) | 1 | 10 |

**Appendix 3: Demographics and specialities of stakeholders who declined to participate.**
